# The impedance analysis of small intestine fusion by pulse source

**DOI:** 10.1515/biol-2020-0082

**Published:** 2020-11-03

**Authors:** Yifan Wang, Kefu Liu, Xiaowei Xiang, Caihui Zhu, Hao Wang

**Affiliations:** Department of Light Sources and Illuminating Engineering, and Academy for Engineering&Technology, Fudan University, Shanghai, 200433, China

**Keywords:** small intestine fusion, pulse source, burst pressure, impedance

## Abstract

The radiofrequency-induced intestine fusion has been widely studied as an alternative for traditional suture in surgery, but fusion quality cannot be evaluated directly. Impedance measurement can evaluate fusion quality, but the relation between impedance and the fusion quality needs optimization for best results. The present study reports the optimum resistance of small intestine fusion. As the feedback signal, resistance was considered the indicator of the fusion completion for the device design of intestine fusion and an in-depth study of microstructure change. A self-design pulse source was used for the small intestine fusion with adjustable voltage, duty ratio, frequency and output time. A frequency of 440 kHz was set, whereas voltage, output time and compression pressure (CP) of the small intestine were independent variables. Different conditions of voltage, CP and time were investigated for achieving the highest burst pressure (BP) measured with a pressure gauge and a peristaltic pump. Each parameter of the equivalent circuit model was calculated by an experimental waveform. Hematoxylin–eosin staining of fusion samples was used for assessing the quality of fusion. The real-time current was measured and recorded during the fusion for the calculation of capacitance and resistance. The highest BP of 38.9 mmHg was achieved with a CP of 900 kPa, a voltage of 50 V and a time of 5 s. Finally, an optimum extracellular resistance range of 61.0–86.2 Ω was found as the optimum resistance for the end of fusion, thus indicating automatic fusion with the best fusion quality.

## 1 Introduction

The principles of radiofrequency (RF)-induced intestine fusion have been investigated for optimization in various areas [1,2,3,4,5,6,7]. In most intestinal cancer surgery, the success of the surgery was dependent on the integrity of the anastomoses in the inflammation phase [8]. Hand suturing was the main method for intestinal anastomoses in the past. However, bleeding and leaks with potential inflammation reduce the success rate of the operation, and the long suturing time increases the operation risk. After the emergence of the laparoscopic surgery, stapling has become the main method for intestinal anastomoses as the convenience accelerates the surgical procedure and the reliability reduces bleeding and leaks. Nowadays, stapling has been applied in large scale in the intestinal surgery, but the potential for failure exists because of the technical shortcomings and the low degree of surgeons’ proficiency. Surgeons even have to do suture after stapling to make sure of the reliability. The high price of stapling also increases the surgery cost [9]. Throughout the years, there have been various attempts to explore alternative anastomotic methods based on RF tissue fusion as it has the following natural advantages: it does not leave foreign material that may induce inflammation and lead to infection, short operation time and low cost.

In 2007, LigaSure Anastomotic Device was developed and used for the intestinal tissue of porcine (four pigs, two anastomoses each), and all seals were macroscopically intact both immediately after creation and healing at the 7th postoperative day. This result confirms the feasibility to create experimental intestinal anastomoses using an RF power source [10]. In 2010, the burst pressure (BP) was used to evaluate the quality of colonic anastomoses *in vitro*. An optimal interval of compression pressure (CP = 1.125 N/mm^2^) with respect to a high amount of BP was detected. There is still a need for further studies exploring the main effects and interactions of tissue and process parameters to the quality of the fusion site [11]. In 2013, the study showed that both bipolar RF energy and optimal CPs were needed to create strong intestinal seals. This finding suggests that RF fusion technology can be effectively applied for bowel sealing and may lead to the development of novel anastomosis tools [12]. In 2014, a novel concave–convex electrode for colonic anastomoses by RF tissue fusion was proposed to reduce the thermal damage essential for anastomotic healing and was verified effectively [13].

Besides the macroscopic aspects, some researchers proposed optic measurements for diagnosing the quality of anastomoses and exploring the insight mechanism. In 2008, real-time optical measurements were applied to improve the understanding of the tissue modifications induced by RF fusion. An algorithm was proposed based on the measurement of the absolute transmittance of the tissue, making use of the modified Beer–Lambert law. Optical measurements show considerable potential as a modality to investigate the process of RF fusion and as a feedback to control RF delivery in real time so that optimal transformations were achieved [14]. In 2014, the first optical Raman spectroscopy study on porcine small intestine was proposed *in vitro*. This study provides direct insights into tissue constituent and structural changes on the molecular level, exposing spectroscopic evidence for the loss of distinct collagen fiber rich tissue layers as well as the denaturing and restructuring of collagen cross-links post RF fusion. These findings open the door for more advanced optical feedback-control methods and characterization during heat-induced tissue fusion, which will lead to new clinical applications of this promising technology [15].

Great progress has been made in the RF intestinal fusion field. However, the aforementioned studies do not provide a direct method for evaluating the quality of fusion in a clinical application. The most important point was how to create an automatic procedure for anastomoses and how to verify the accomplishment of the procedure. As in the clinical application, measuring BP was not possible; optic measurements need extra devices, which increase the complexity of surgery. In fact, the impedance measurement was a feasible method to evaluate the quality of fusion, but there were few reports to study the relation between impedance and the quality of fusion. In this study, the optimum resistance was proposed for the accomplishment of small intestine fusion which reaches high BP based on the large-scale experiments *in vitro*. Fusion with different voltage levels, CPs and fusion time periods has been verified.

## 2 Materials and methods

### 2.1 Pulse source

A self-design constant voltage pulse source was used for the experiment. The bipolar square wave was generated by a full-bridge converter. The voltage value, duty ratio, frequency and output time were all adjustable. In this study, the duty ratio was set at 72.7% and the frequency was set at 440 kHz. The output voltage was measured by a differential probe RP2015D made by Rigol and an oscilloscope 6403D made by PicoScope. The rise time (10–90%) from negative 50 V to positive 50 V was 24 ns, and the fall time (10–90%) was 45 ns under the same condition for each pulse. In this study, different voltage values from 20 to 80 V and output time periods of 5, 10 and 15 s were applied to the small intestine.

### 2.2 Compression device and BP measurement

**Figure 1:**
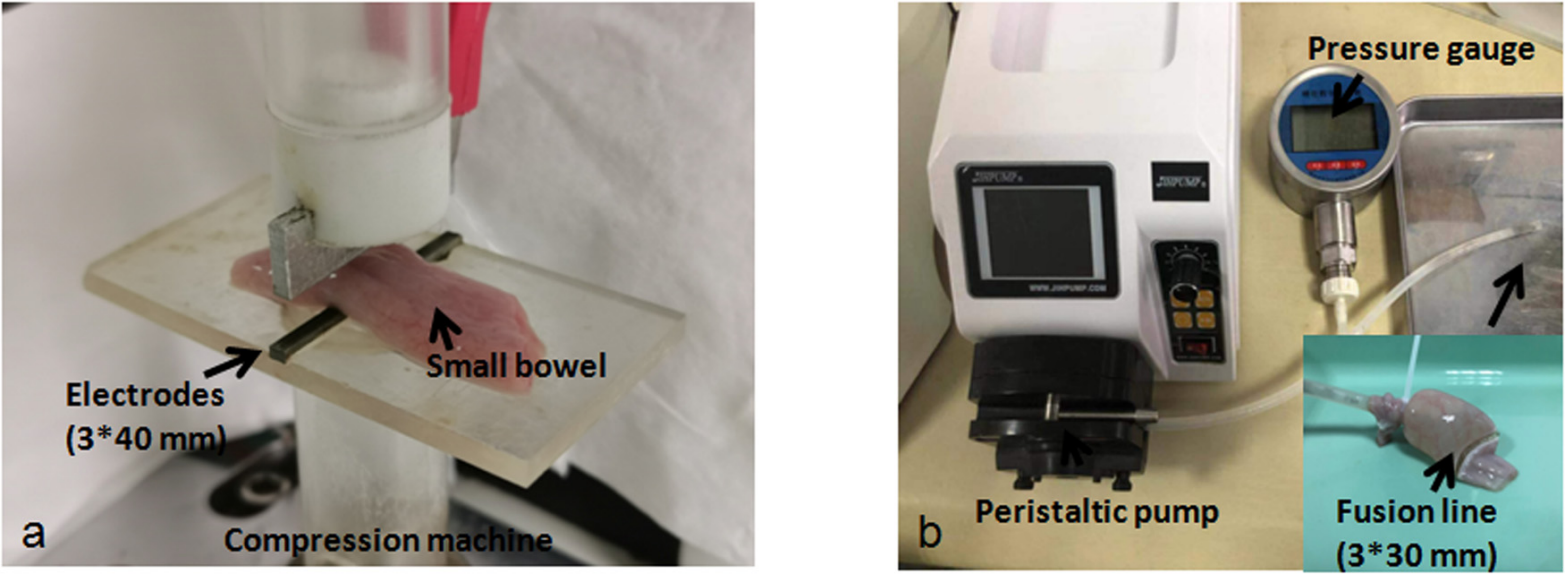
(a) Compression machine. (b) Peristaltic pump, pressure gauge and BP measurement.

The compression machine ZQ-990A-1 was made by Zhiqu Precision Instruments with a pressure from 0 to 200 N (Figure 1a). The applied pressure can automatically adapt to the varying thickness during the fusion for keeping the pressure constant. The small intestine was placed on the bottom clamp, and then, the top clamp was pushed down for clamping small intestine. After several seconds, the pressure was constant, then the pulse source outputs the voltage. In this study, the six CPs were 36, 45, 54, 63, 72 and 81 N and the fusion time was 5, 10 or 15 s. The electrode material was nickel–chromium alloy with a surface size of 3 × 40 mm. As the width of the small intestine was around 30 mm, the fusion surface size was 3 mm × 30 mm. Intensity of pressures was calculated by CP/fusion surface size, which were 400, 500, 600, 700, 800 and 900 kPa.

The peristaltic pump BT-100CA was made by Jihpump with a flow rate from 0.07 to 79 mL/min (Figure 1b). In this study, the flow rate was set at 5 mL/min. The pressure gauge YK-100 was made by Shileke Technology with a measuring range of 0–1,500 mmHg and an accuracy of 0.4 mmHg (Figure 1b). The three terminals of the T-tube were connected separately to the pressure gauge, peristaltic pump and one end of the small intestine (another end of the small intestine has been sealed by fusion). The BP was defined as the maximum pressure measured during infusion. Once the fused anastomotic line started to leak, the pressure began to drop.

### 2.3 Impedance measurement

Pearson current monitor 4100 and oscilloscope 6403D were used for measuring the current. As the voltage was constant, impedance was calculated by the voltage/current.

### 2.4 Preparation of fresh small intestine

A complete porcine small intestine was harvested from pigs at a slaughterhouse. After cleaning, the small intestine was immersed in 0.9% saline and delivered at 0–4°C to the laboratory for the experiments. Before the experiment, a secondary cleaning was applied to the small intestine. The small intestine was then cut into 70 mm segments and immersed in 0.9% saline until fusion. All the prepared small intestines were used for fusion experiments within 24 h after harvest. Mucosa-to-mucosa fusions were formed on the porcine small intestine segments *in vitro* by the pulse source mentioned earlier.

### 2.5 Experimental process

**Figure 2:**
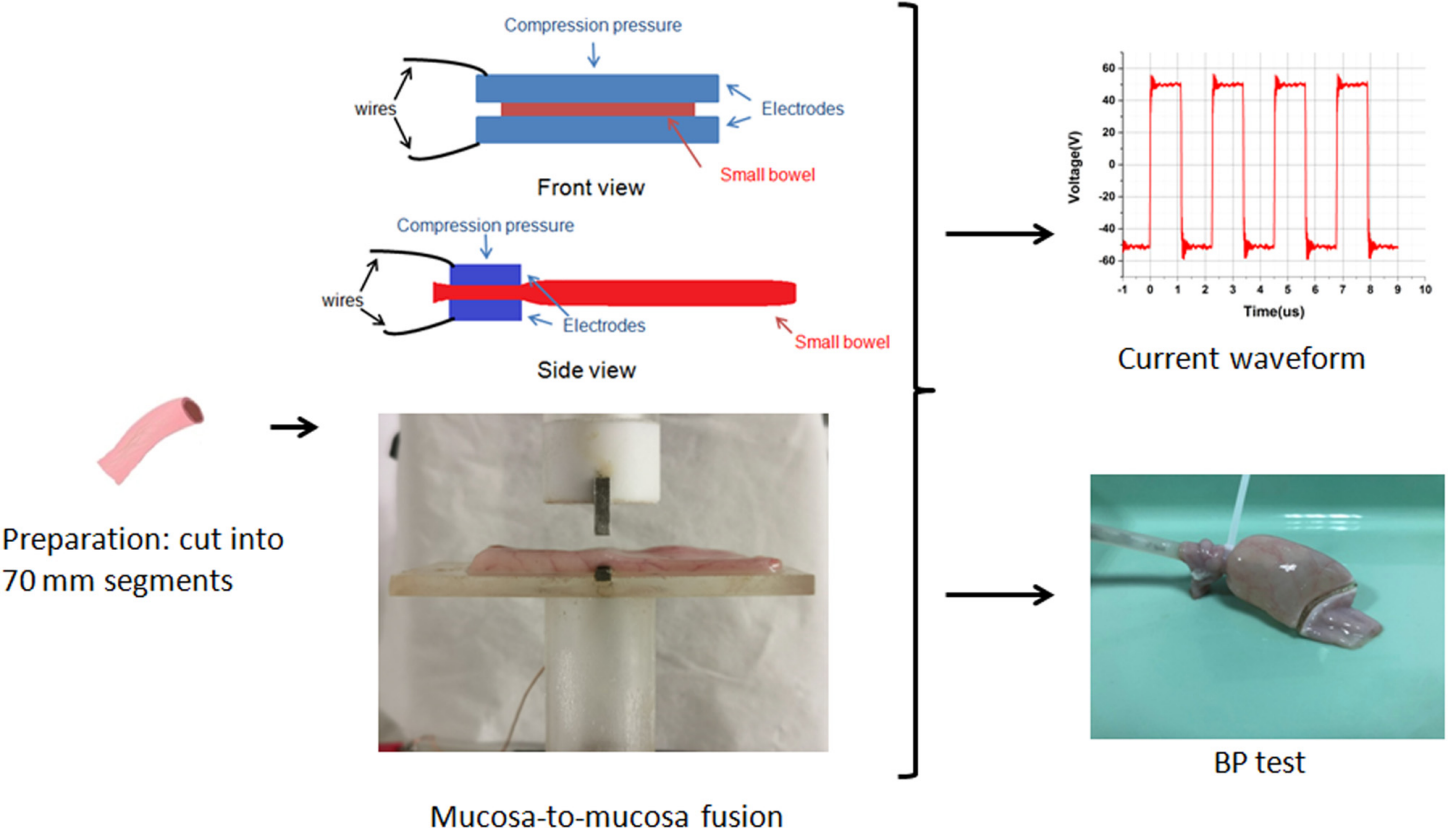
Experimental process.

The small intestines were harvested from the pigs and cleaned at the slaughterhouse. After being delivered to the laboratory, the small intestines were cut into 70 mm segments. Each small intestine was placed on the bottom clamp, then the pressure machine began to push the top clamp down for applying pressure. This process usually took several seconds, and then, the pulse source was turned on for generating pulse square voltage, which was transferred by wires to clamps. The oscilloscope 6403D recorded the complete waveform of the current during the fusion. The small intestines were tested for BP after the fusion (Figure 2).

### 2.6 Histology

Fused small intestines were fixed in formalin immediately after fusion. The tissue was processed in paraffin wax and prepared in slices, then cut transversely to the seal and stained with hematoxylin–eosin (H&E). The prepared intestinal slices were mounted on the stereo microscope (SZ680; Optics, China) and were visualized and imaged.


**Ethical approval:** The conducted research did not require ethical approval because the animal material was taken from the slaughterhouse.

## 3 Results

### 3.1 BP

Due to the existence of the biological tissue heterogeneity and the measuring error, the BPs of the adjacent segments were usually different. In order to get the accurate data with less sampling error, each BP was the average value of the ten experiments carried out under the same conditions. For example, the BP was 28.1 ± 2.43 mmHg (average value ± variance value) under the condition of 50 V, 700 kPa and 5 s. The highest value and the lowest value of the ten values were removed while calculating the average value for less sampling error.

**Figure 3:**
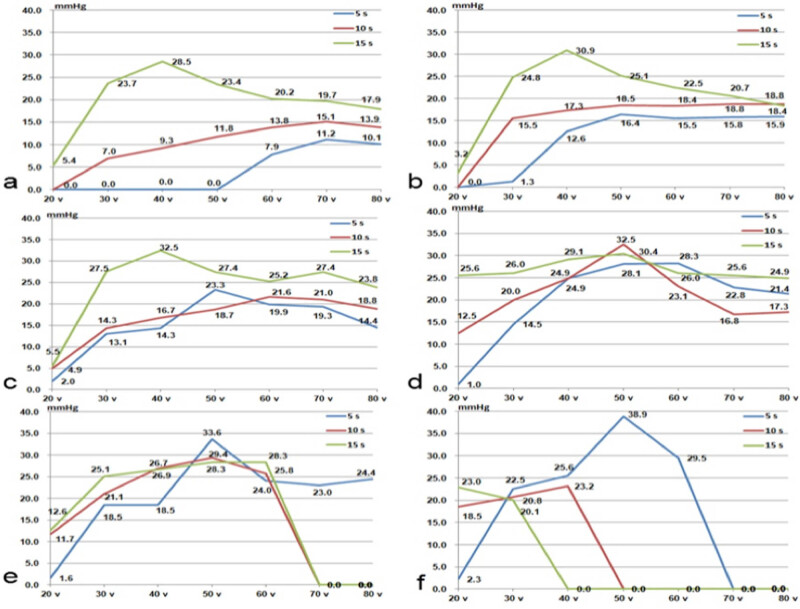
BP of the small bowels with different CP, voltage and time. The horizontal axis is voltage and the vertical axis is BP for each graph; the blue line is the BP with 5 s, the red line is the BP with 10 s and the green line is the BP with 15 s: (a) CP = 400 kPa; (b) CP = 500 kPa; (c) CP = 600 kPa; (d) CP = 700 kPa; (e) CP = 800 kPa; and (f) CP = 900 kPa.

In this study, a total of six groups of small intestines were tested for BP, including 106 BP values, which means 1,060 segments were tested for BP (Figure 3).

For CP less than 400 kPa, the BP was lower than 15 mmHg, and most fusions failed. So the aforementioned data only included the fusion with CP greater than or equal to 400 kPa. CP, voltage and fusion time were all factors influencing BP. For relatively low CP, voltage and relatively short fusion time, the fusion failed; for relatively high CP, voltage and relatively long fusion time, the temperature was too high for optimum fusion, so the fusion also failed. In each graph, the BPs usually increased at first and then decreased except 10 and 15 s in Figure 3a and b. For CP of 400 or 500 kPa, time of 10 or 15 s, the highest BP needed a high voltage of 70 or 80 V. For a high CP of 900 kPa and a long fusion time of 15 s, the highest BP needed only a voltage of 20 V. With the increase of CP, the optimum time decreased from 15 to 5 s.

**Figure 4:**
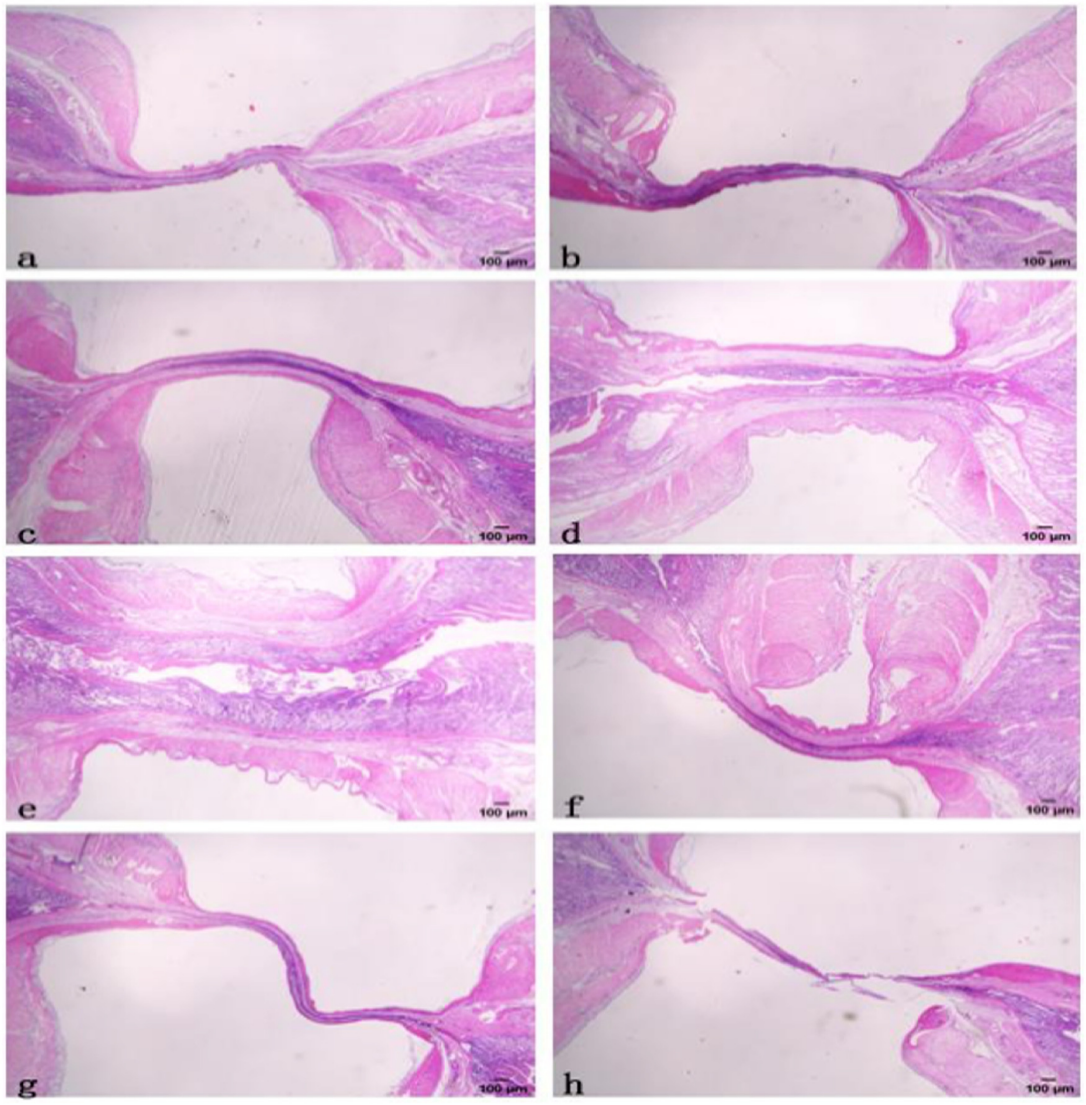
Transverse slice and H&E staining of fusion samples of 50 V and 5 s for: (a) CP = 900 kPa; (b) CP = 700 kPa; (c) CP = 500 kPa; (d) CP = 300 kPa; (e) CP = 100 kPa; (f) transverse slice and H&E staining of fusion samples of 30 V, 5 s and CP = 700 kPa; (g) transverse slice and H&E staining of fusion samples of 70 V, 5 s and CP = 700 kPa; (h) transverse slice and H&E staining of fusion samples of 70 V, 5 s and CP = 900 kPa.

The transverse slice and H&E staining of fusion samples are shown in Figure 4. With the increase of CP, the width of the fusion line decreased and the tightness of the fusion line increased (Figure 4a–e). With the CP of 100 and 300 kPa, there was an obvious gap in the fusion line (Figure 4d and e). So the BPs of fusion with 300 kPa or below were relatively too low and not included in the above discussion. In Figure 4b, f and g, the fusion time and CP were the same; with the increase of voltage, the width of the fusion line decreased and the tightness of the fusion line increased. However, the high level of voltage and CP was adverse to the fusion, as shown in Figure 4h. The fracture at the fusion line indicated that the fusion failed, which was also consistent with the BP results.

### 3.2 Impedance analysis

**Figure 5:**
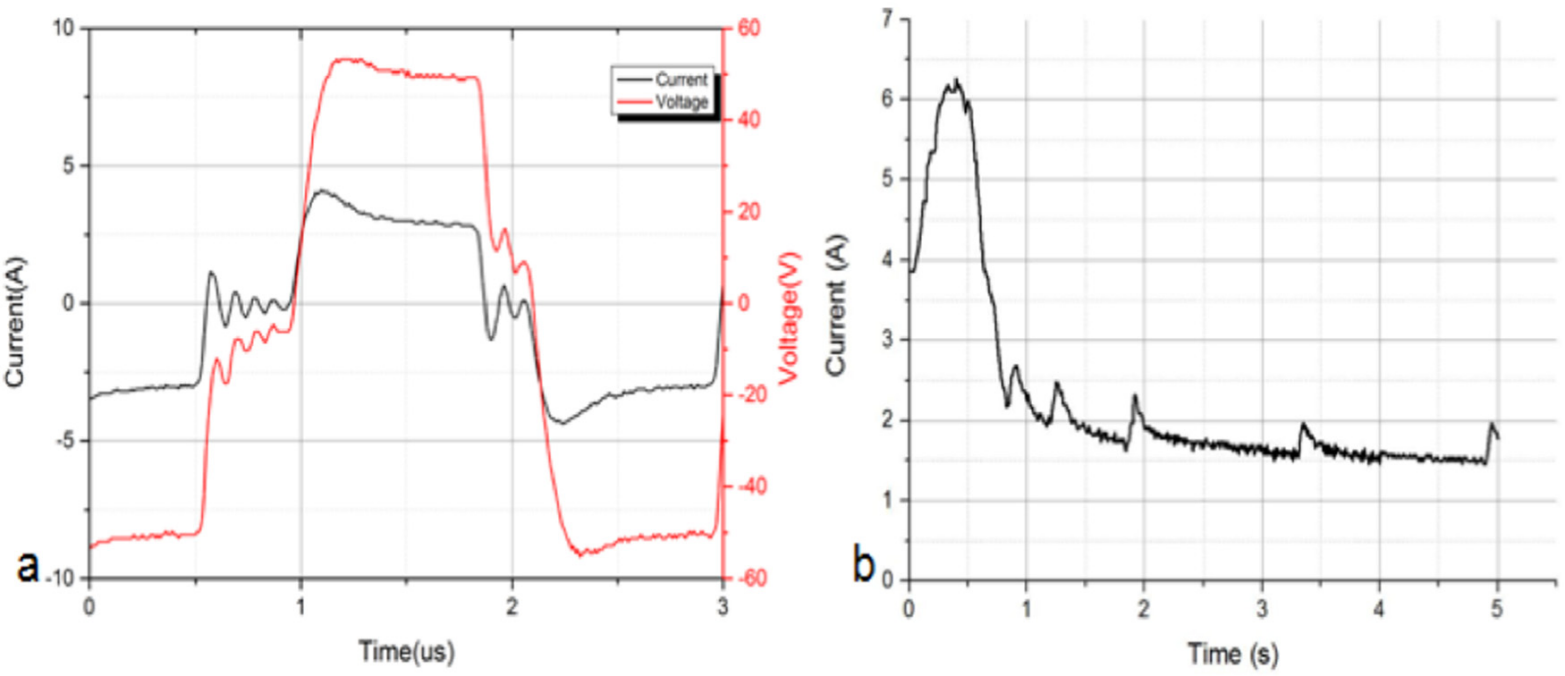
(a) Current and voltage waveform of 700 kPa, 50 V and 5 s at time 0.1 s. (b) The peak currents of total positive pulses of 700 kPa, 50 V and 5 s.

Figure 5a shows the current waveform at time 0.1 s under 700 kPa, 50 V and 5 s; the voltage was square waveform with an amplitude of 50 V; the current was an approximate square waveform with an overshoot at the rising edge. This was because a part of the current was used for membrane capacitors charging at the beginning. Figure 5a shows one complete pulse with a pulse width of ∼1 µs. As the frequency of voltage was 440 kHz, the total number of pulses was 4,400k in the fusion time of 5 s. The number of positive pulses was 2,200k. The peak currents of these positive pulses were collected and shown in Figure 5b. The beginning current was 3.86 A, so the impedance was 12.95 Ω. The maximum current was 6.26 A, so the minimum impedance was 7.99 Ω, after which the current began to decrease. The final current was 1.77 A, and the impedance was 28.25 Ω, excluding the oscillation. The current waveforms of 10 and 15 s were not recorded, as the depth of the oscilloscope 6403D was 1 G, which means that for the current waveform of 5 s, the sampling rate was around 19 ns. The increased time will increase the sampling rate and decrease the accuracy of the measurement; thus, the data were incomparable. Another reason was that the slope of the current after 5 s did not change much.

**Figure 6:**
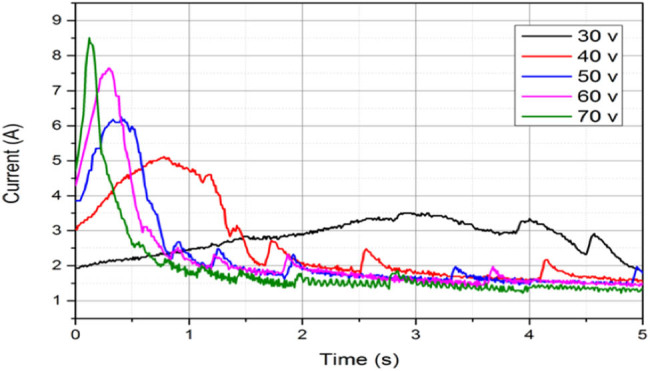
Impedance of different voltages with current 30–70 V.

**Figure 7:**
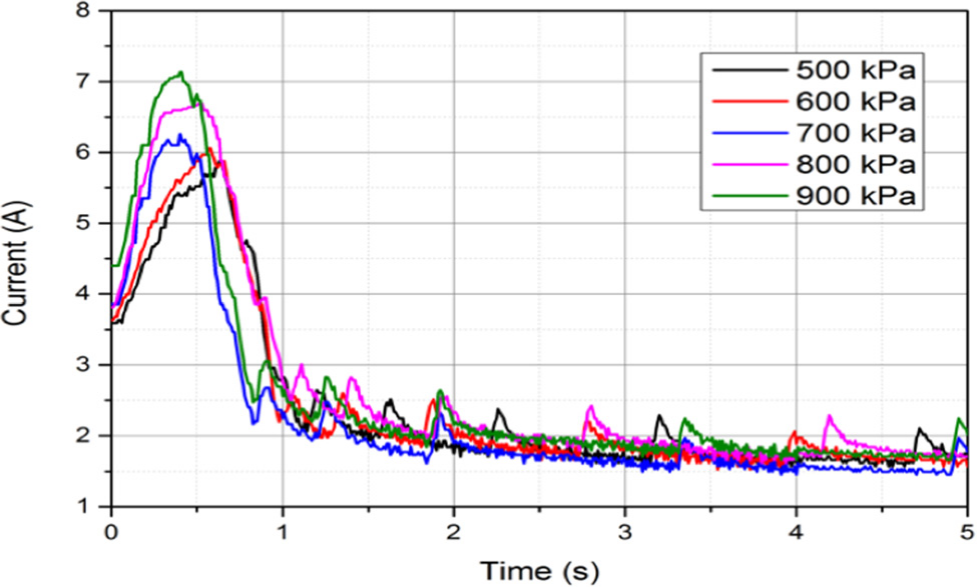
Impedance of different CPs with current 500–900 kPa.

Figure 6 shows the impedance of different voltages with current 30–70 V. The initial impedance and the minimum impedance decreased as the voltage increased. Meanwhile, the time between initial impedance and minimum impedance decreased as the voltage increased. With higher voltage, the change of tissue internal suture accelerated. Figure 7 shows the impedance of different CPs with current 500–900 kPa. The initial impedance and the minimum impedance increased as the CP increased. This was because the increase of CP increased the contact between electrodes and tissue, and between the internal cells in tissue, then the impedance decreased.

**Figure 8:**
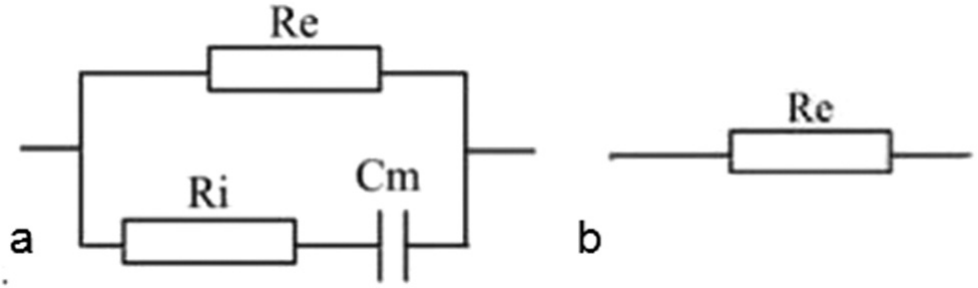
Equivalent circuit model of the tissue when the frequency was extremely high (a) and low (b).

2R-1C was the common model to analyze the bioimpedance (Figure 8a). Extracellular and intracellular space can be modeled as resistors (*R*_e_ and *R*_i_, respectively), and the cell membrane can be modeled as capacitor *C*_m_.

**Figure 9:**
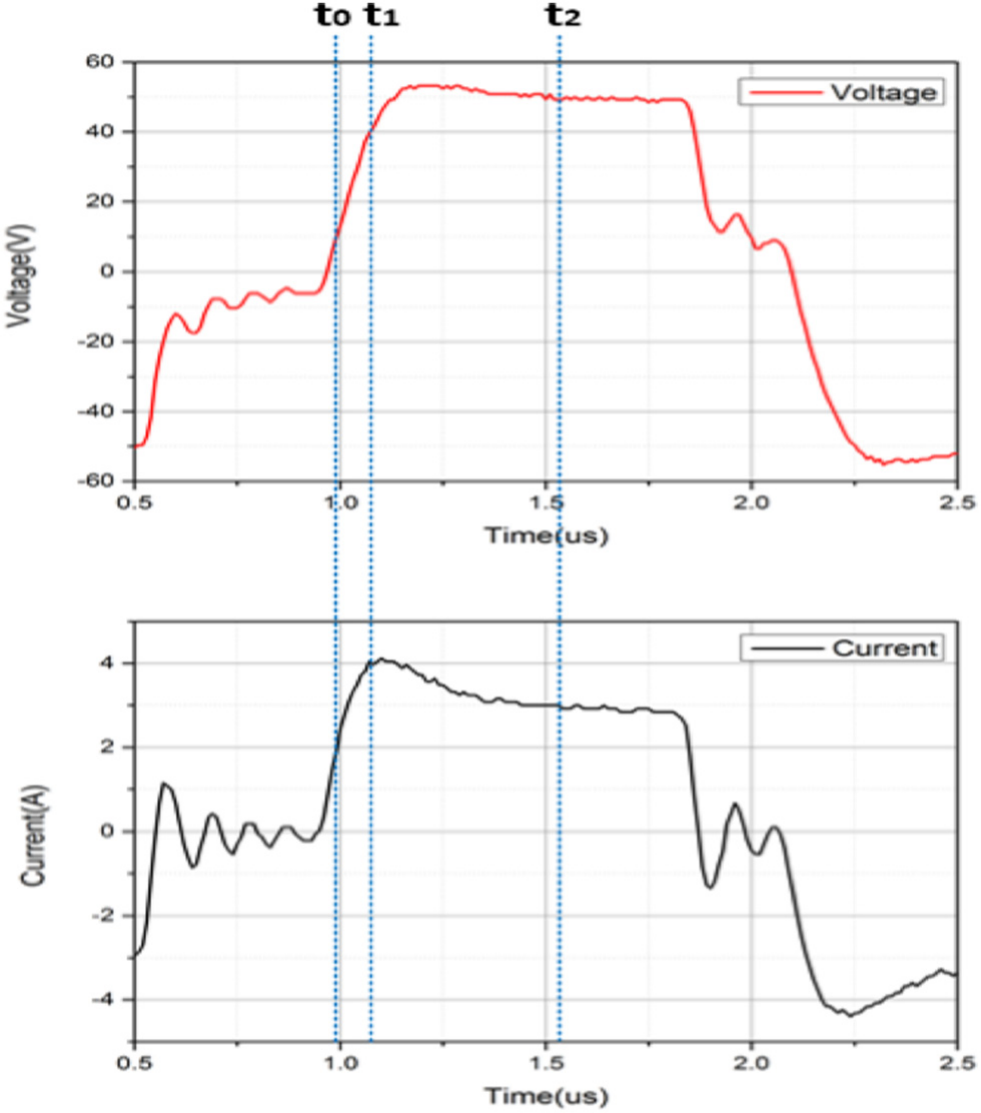
Amplification of current and voltage waveforms under 700 kPa, 50 V and 5 s at time 0.1 s.

At low frequencies or DC, the membrane capacitors hindered the current, and most of current flow around the cells was not able to penetrate into cells. Thus, the model only consisted of *R*_e_ (Figure 8b). Figure 9 shows the amplification of the current waveform of Figure 5b at different times. The complete pulse of voltage starts at the time 0.91 s and ends at 2.10 s; time *t*_2_ is equal to 1.54 s, which is (0.91 + 2.10)/2 s. The voltage at *t*_2_ is set as the amplitude for this pulse. The voltage at time *t*_0_ (0.98 s) is 10% of the amplitude and the voltage at time *t*_1_ (1.10 s) is 90% of the amplitude.

The current overshoot is obvious in Figure 9 because of the existence of the capacitor. For a circuit of paralleled relatively smaller capacitance and relatively bigger resistance, a part of the current was on the capacitance at the beginning. With the increase of current on the capacitance, the current on the resistance also increased. There was a time that the current of capacitance began to decrease, and at last, the current on the capacitance was zero and the whole current was on the resistance. So there was a peak current at the beginning for each pulse due to the existence of the capacitance, and the overshoot current was actually a charging process. With lower resistance, the current was higher and enough for the capacitor charging.

**Figure 10:**
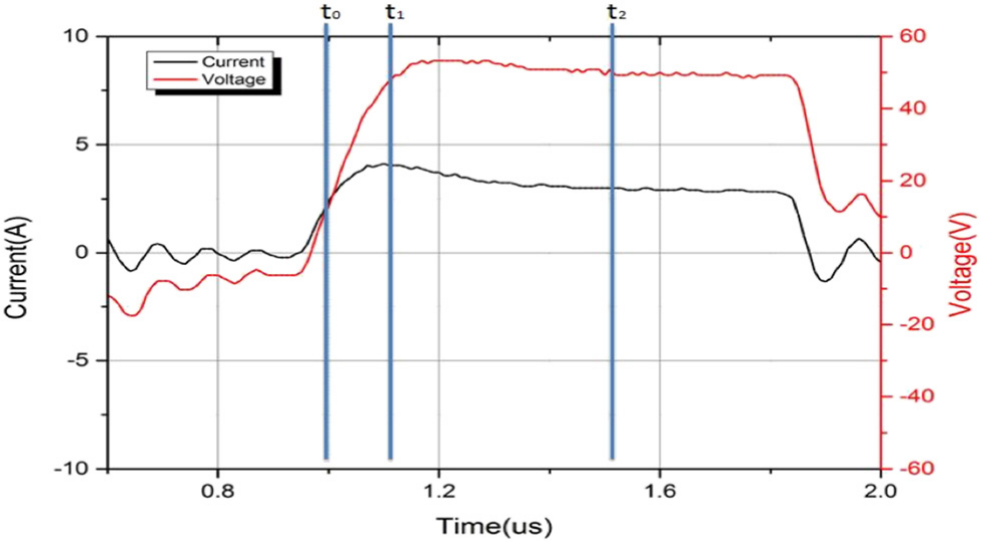
Amplification of current and voltage waveforms under 700 kPa, 50 V and 5 s at time 0.1 s.

In Figure 8, the equivalent circuit model of the tissue was explained. According to the voltage and current waveform, we could calculate the values of *R*_e_, *R*_i_ and *C*_m_. For example, in Figure 5a, we calculated the values of *R*_e_, *R*_i_ and *C*_m_. The amplification of Figure 5a is shown in Figure 10. First, we assumed that the values of *R*_e_, *R*_i_ and *C*_m_ did not change in one single pulse, which means in Figure 10 the values of *R*_e_, *R*_i_ and *C*_m_ did not change.

At *t*_2_, the voltage and current were stable and the voltage on the tissue was 50 V and equal to the voltage of source, which means the capacitor was charged to the 50 V and all current flowed through *R*_e_. So the *R*_e_ could be calculated by voltage divided by current at this time. At time *t*_0_ and *t*_1_, the capacitor was under charging progress. We assumed that the current flow through *R*_e_ was *i*_1_, and the current flow through *R*_i_ and *C*_m_ was *i*_2_ [see equation (1)].

(1) \begin{array}{l}U={R}_{\text{e}}\cdot {i}_{1}={R}_{\text{i}}\cdot {i}_{2}+\frac{\int {i}_{2}\cdot \text{Δ}t}{{C}_{\text{m}}}\\ i={i}_{1}+{i}_{2}\end{array}

In equation (1), as *R*_e_ was already calculated, so *i*_1_ was calculated by \tfrac{U}{{R}_{\text{e}}}, then *i*_2_ was known. Using the data at *t*_0_ and *t*_1_, we calculated the value of *R*_i_ and *C*_m_; the value of *R*_i_ was 1.90 Ω and the value of *C*_m_ was 5.02 nF. The values of *R*_e_, *R*_i_ and *C*_m_ in each figure of Figure 10 were calculated and are shown in Table 1.

**Table 1:** Values of *R*_e_, *R*_i_ and *C*_m_ of 700 kPa, 50 V and 5 s at time 0.1 s, 0.4 s and 3.5 s

Time (s)	*R*_e_ (Ω)	*R*_i_ (Ω)	*C*_m_ (nF)
0.1	17.12	1.90	5.02
0.4	15.80	0.70	4.03
3.5	64.94	63.28	1.89

**Figure 11:**
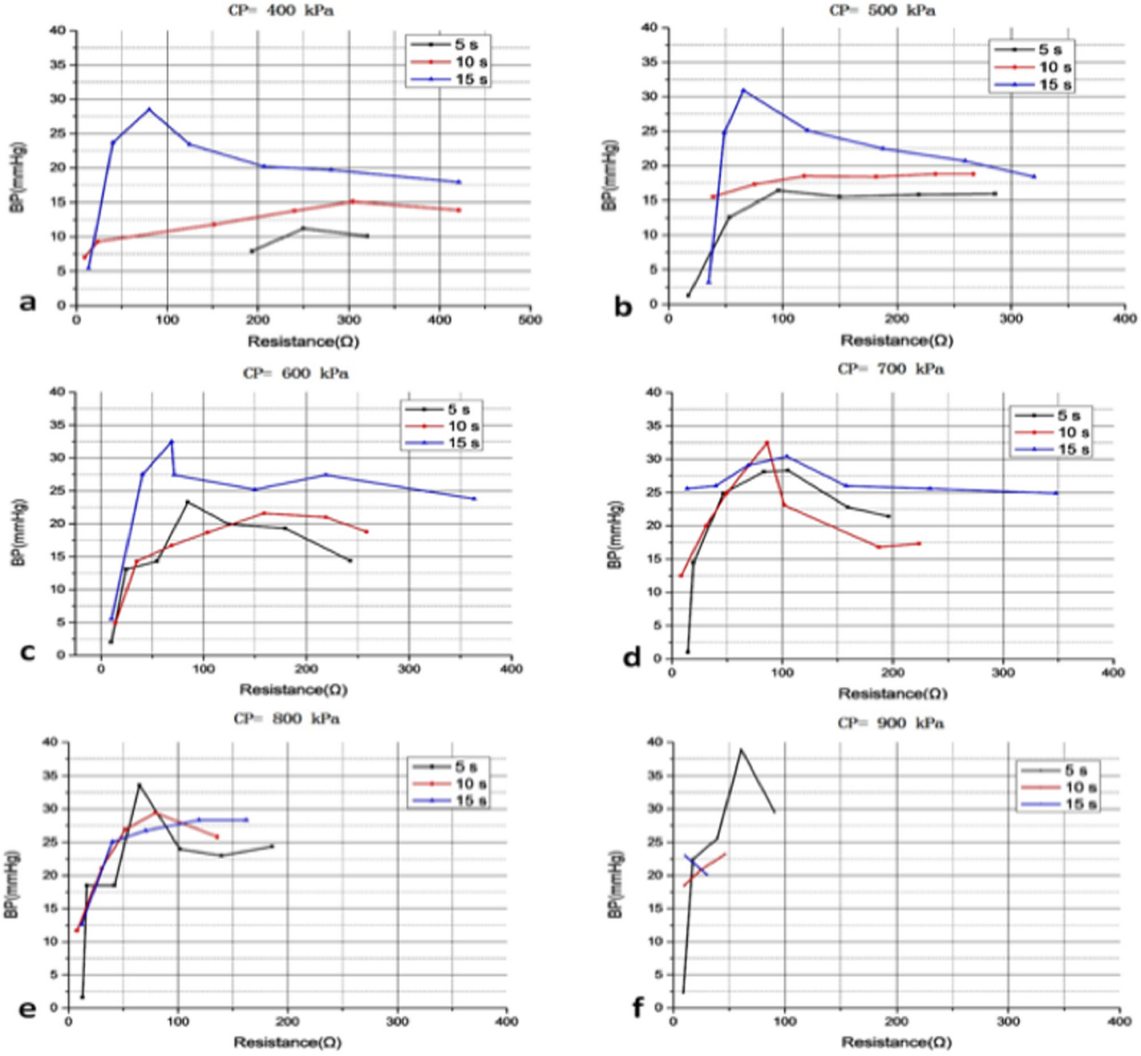
Relation of BP and *R*_e_ under different conditions: (a) CP = 400 kPa; (b) CP = 500 kPa; (c) CP = 600 kPa; (d) CP = 700 kPa; (e) CP = 800 kPa; and (f) CP = 900 kPa. In each graph, the black line is the result of 5 s, the red is the result of 10 s and the blue line is the result of 15 s. For each dot on the same line, the voltage was different, and the resistance increased as the voltage increased.

The *R*_e_ at the end of fusion of each condition was extracted and recorded for each experiment. Figure 11 shows the relation of BP and *R*_e_ under different conditions. For a CP of 400 kPa, the highest BP was 28.5 mmHg with a resistance of 80.0 Ω; for a CP of 500 kPa, the highest BP was 30.9 mmHg with a resistance of 65.6 Ω; for a CP of 600 kPa, the highest BP was 32.5 mmHg with a resistance of 69.0 Ω; for a CP of 700 kPa, the highest BP was 32.5 mmHg with a resistance of 86.2 Ω; for a CP of 800 kPa, the highest BP was 33.6 mmHg with a resistance of 64.9 Ω; and for a CP of 900 kPa, the highest BP was 38.9 mmHg with a resistance of 61.0 Ω. For each line, the resistance increased as the voltage increased, so the voltage was the lowest for the first one on the left and the highest for the first one on the right on each line.

## 4 Discussion

In this study, the BP of the small intestine fusion was measured based on large-scale experiments. Voltage, CP and fusion time were all factors affecting the BP. The maximum BP was 38.9 mmHg when the parameters were 50 V, 900 kPa and 5 s. The high fusion quality needed appropriate parameters. Low voltage, CP or short fusion time could not provide high seal strength; high voltage, CP or long fusion time might have caused thermal damage to the small intestine and was actually adverse to fusion. Small intestine fusion needed suitable parameters. The maximum BP was 38.9 mmHg when the parameters were 50 V, 900 kPa and 5 s. In 2013, a research group reached a maximum BP of 27.56 mmHg under the condition of 150 kPa. For other parameters, the study only mentioned that the frequency was 473 kHz [11]. For other similar studies, only one parameter was usually discussed. The combined influence of voltage, CP and time was analyzed for the first time. The optimum fusion quality was the combined action of CP, voltage and fusion time.

The transverse slice and H&E staining of fusion samples were shown next. This result was matched to the BP result: the tight fusion line with appropriate parameters. The tissue structure from a microscopic aspect explained the relation between BP and parameters. Low voltage or CP could not provide a tight fusion line; high voltage or CP induced fracture at the fusion line and made the fusion fail.

The current during the fusion was recorded, and a dynamic capacitance was calculated for the first time in the tissue fusion field. In Figure 5, current waveform of 700 kPa, 50 V and 5 s was recorded. However, the curve was not smooth. When the fusion began, the small intestine deformed, so the top clamp accelerated, moving down for keeping constant pressure. In a very short time, the CP was lower than the preset value. When the pressure reached the preset value, the speed of the top clamp began to decrease, and in this very short time, the actual pressure was greater than the preset value as the top clamp was still moving down. Lower pressure decreased the tightness of the small intestine, so the conductive channel was weakened and the current decreased; greater pressure increased the tightness of the small intestine, so the conductive channel was enhanced and the current increased. If the response speed was quick enough, the current should have been a smooth curve. The current waveforms of 10 and 15 s were not recorded, as the depth of the oscilloscope 6403D was 1G, which means that for a current waveform of 5 s, the sampling rate was around 19 ns. The increased time should increase the sampling rate and decrease the accuracy of the measurement; thus, the data were incomparable. Another reason was that the slope of the current after 5 s did not change much.

In Figures 6 and 7, the influences of voltage and CP were compared. The changes of impedance under different voltages and CPs were discussed. Then, the values of *R*_e_, *R*_i_ and *C*_m_ during fusion were calculated by an experimental waveform. The *R*_e_ and *R*_i_ decreased at the beginning time and then increased, and the *C*_m_ decreased during the whole fusion time. There might be two reasons for the capacitors to decrease. The first may be that many cells ruptured under the voltage and CP, so a large number of paralleled membrane capacitances disappeared; the second was that the cells formed regular arrays in the direction of the electric field, so the paralleled cells became series cells which decreased the total capacitance. The decrease of *R*_e_ and *R*_i_ at the beginning time might be because the electrical field on the tissue promoted ion migration, so the electrical conductivity increased, and then, mass evaporation of water decreased the electrical conductivity and increased the resistance. The extra microscopic experiment was essential for explaining the process in the tissue.

In Figure 11, even *R*_e_ at the end of the fusion differs a lot; from 7.6 to 421.1 Ω, the optimum resistance was concentrated. Based on this large-scale experiment, there was an optimum resistance range of 61.0–86.2 Ω. With a preset voltage and CP, the resistance decreased at the beginning and then increased as the time increased. Regardless of the value of voltage and CP, the small intestines would be sealed at a time with the optimum resistance. For pulse source design, the current at this moment would be the best feedback signal at the end of fusion.

This was a macroscopical result for the small intestine fusion, and the microscopic change needs to be verified by more experiments to explain this optimum resistance. What kind of chemical reaction or physical reaction occurred in cells or tissues during the fusion needs more study. In our view, the small intestine will be in the optimum fusion quality with a certain microscopic structure, and *R*_e_ in this condition should also be a certain value. The microscopic studies need additional experiments to be verified.

## 5 Conclusion

In this study, a total of six groups of small intestines were tested for BP, including 106 BP values, which means 1,060 segments were tested for BP. The combined influence of voltage, CP and time was discussed. The highest BP was 38.9 mmHg with 900 kPa, 50 V and 5 s. The transverse slice and H&E staining of fusion samples explained the relation between BP and parameters from a microscopic aspect. Then, the current during the fusion was measured and discussed for the impedance analysis. The values of *R*_e_, *R*_i_ and *C*_m_ were calculated by an experimental waveform. The change in them during fusion was discussed. Finally, an optimum *R*_e_ range of 61.0–86.2 Ω was proposed as the optimum resistance for the end of fusion, which will be an indicator for automatic fusion with the best fusion quality. The quality of fusion was a result of a combined influence of CP, voltage and time. The optimum extracellular resistance between 61.0 and 86.2 Ω was proposed for the highest BPs and automatic fusion. The findings of this study may provide useful insights for a quality intestinal fusion with higher safety and accuracy while employing this easy-to-use method of impedance measurement.
